# Outcome of extra mucosal single interrupted layer (EMSIL) ileo-ileal anastomosis in urinary diversions during open radical cystectomy

**DOI:** 10.1186/s12301-023-00357-3

**Published:** 2023-05-08

**Authors:** Abdul Rouf Khawaja, Mujahid Ahmad Mir, Arshi Beg, Shahid M. Baba, Mohammad Saleem Wani, Arif Hamid

**Affiliations:** 1 Department of Urology and Kidney Transplant Unit, Sher-i-Kashmir Institute of Medical Sciences, Soura, Srinagar, Jammu And Kashmir 190011 India; 2 Department of Pathology, Tata Memorial Hospital, Mumbai, India

**Keywords:** Bladder cancer, Cystectomy, Ileal-conduit, Orthotopic-neobladder, Perioperative complications

## Abstract

**Background:**

We intended to assess the outcome of extra-mucosal single interrupted layer ileo-ileal anastomosis for bowel re-approximation in open radical cystectomy with urinary diversions.

**Methods:**

This is a prospective study of patients who had extra-mucosal ileo-ileal intestinal anastomosis following radical cystectomy and urinary diversion at our institution from January 2018 to April 2021. Data was collected from patient medical records and analyzed by using SPSS Statistics for Windows version 25.0. Data was expressed as a mean ± standard deviation (SD) or median for continuous variables, whereas frequency and percentage were used to express qualitative data. Operative time and anastomosis time, blood loss, hospitalization duration, and time taken for the return of bowel activity were studied. Perioperative complications were noted down.

**Results:**

Fifty-nine patients were selected for our study. Urinary diversion was achieved in the form of ileal conduit (IC) in 49 patients and orthotopic neobladder (ONB) in 10 patients following radical cystectomy. The mean operative duration was 263.8 ± 48.9 min and the mean anastomosis time was 17.3 ± 5 min. Thirty-eight patients needed blood transfusion (0.97 ± 0.79 units per patient). The mean (± SD) time taken for the return of bowel activity was 84.6 ± 10 h and the average (± SD) post-operative hospital stay was 12.6 ± 3.1 days. There was no anastomotic leak or any other major intestinal complication in any of our patients.

**Conclusions:**

Extra-mucosal single layer ileo-ileal anastomosis for bowel re- approximation is safe and is associated with acceptable and easily managed complications in patients following radical cystectomy and urinary diversion.

## Background

Open radical cystectomy (ORC) is the gold-standard approach for management of muscle invasive and non-muscle invasive bladder cancer that is not amenable to bladder preservation [1]. ORC provides excellent local control and highly accurate pathological staging and grading options [2]. However, it is a major procedure with potential for serious complications, and it is associated with significant morbidity and mortality rates of 26–64% [3], particularly in the early postoperative period.

In addition to slowed bowel motility, which is expected for 24–48 h after any major abdominal surgery, 17–30% of common complications associated with ORC are related to intestinal anastomosis in the early post-operative period. Postoperative ileus and partial bowel obstruction are the most common minor complications reported after ORC, followed by nausea, diarrhea, and the need for nasogastric tube placement or total parenteral nutrition. Postoperative ileus is generally defined as intolerance to oral intake persisting beyond five days postoperatively or by nausea and vomiting accompanied by abdominal distention requiring gut-rest (nothing by mouth, nasogastric tube, or total parenteral nutrition) at any time postoperatively [4]. These outcomes lead to extended hospital stays and increased hospital costs. Major complications include complete bowel obstruction, gastrointestinal bleeding, bowel leakage, and entero-cutaneous fistula. Intense tactile manipulation, prolonged environmental exposure, and altered nutrient supply to the intestinal tract are the main causes of gut-related morbidity following ORC.

Extra-mucosal single interrupted layer (EMSIL) anastomosis for re-approximating bowel is an excellent technique with low complication rate as it causes less tissue necrosis and luminal narrowing. The literature on this technique is quite meager in Indian populations [5–7]. We thus present our experience of EMSIL ileo-ileal anastomosis for bowel continuity following ORC with urinary diversions at our tertiary care hospital.

## Methods

The present study is a prospective observational study approved by the Institutional Ethical Committee of our institution. All participants provided informed written consent, and all patients who underwent ORC with urinary diversion at our hospital between January 2018 and April 2021 were enrolled in our study. Patients underwent the necessary preoperative workup, including complete blood counts, liver and kidney function tests, and posteroanterior chest radiography. For loco-regional disease assessment, contrast-enhanced computed tomography was used in cases of normal kidney function and magnetic resonance imaging without contrast otherwise. The pre-surgery parameters included gender, age, comorbidities, and American Society of Anesthesiologists score.

Urinary diversion IC or ONB was created based upon factors such as kidney function status, disease spread and patient preference. Operative duration, anastomosis time, amount of blood loss, time to normal bowel function return, time to start oral feeding, length of hospital stay, and complications were studied in the perioperative period. Intraoperative blood loss was estimated by visual assessment of surgical sponges, suction canisters, and the operating room environment and discussion between the surgeon and anesthesiologist. Data was collected from patient medical records and analyzed by using SPSS Statistics for Windows version 25.0. Data was expressed as a mean ± standard deviation (SD) or median for continuous variables, whereas frequency and percentage were used to express qualitative data. Mechanical bowel preparation with polyethylene glycol solution was routinely done in all patients. Low molecular weight heparin, pneumatic compression devices, and early mobilization practices were used for deep venous thrombosis prophylaxis.

## Surgical technique and follow-up

All procedures were performed by a single senior surgeon experienced in uro-oncologic surgery. The standard surgery procedure was followed using a midline infra umbilical incision under endotracheal anesthesia in all patients. Radical cystectomy by antegrade approach followed by standard bilateral pelvic lymph node dissection was performed. An ileal conduit (IC) or orthotopic neobladder (ONB) was performed as desired by the patient. Bowel continuity was achieved by hand-sewn EMSIL ileoileal anastomosis in all patients.

**Fig. 1:**
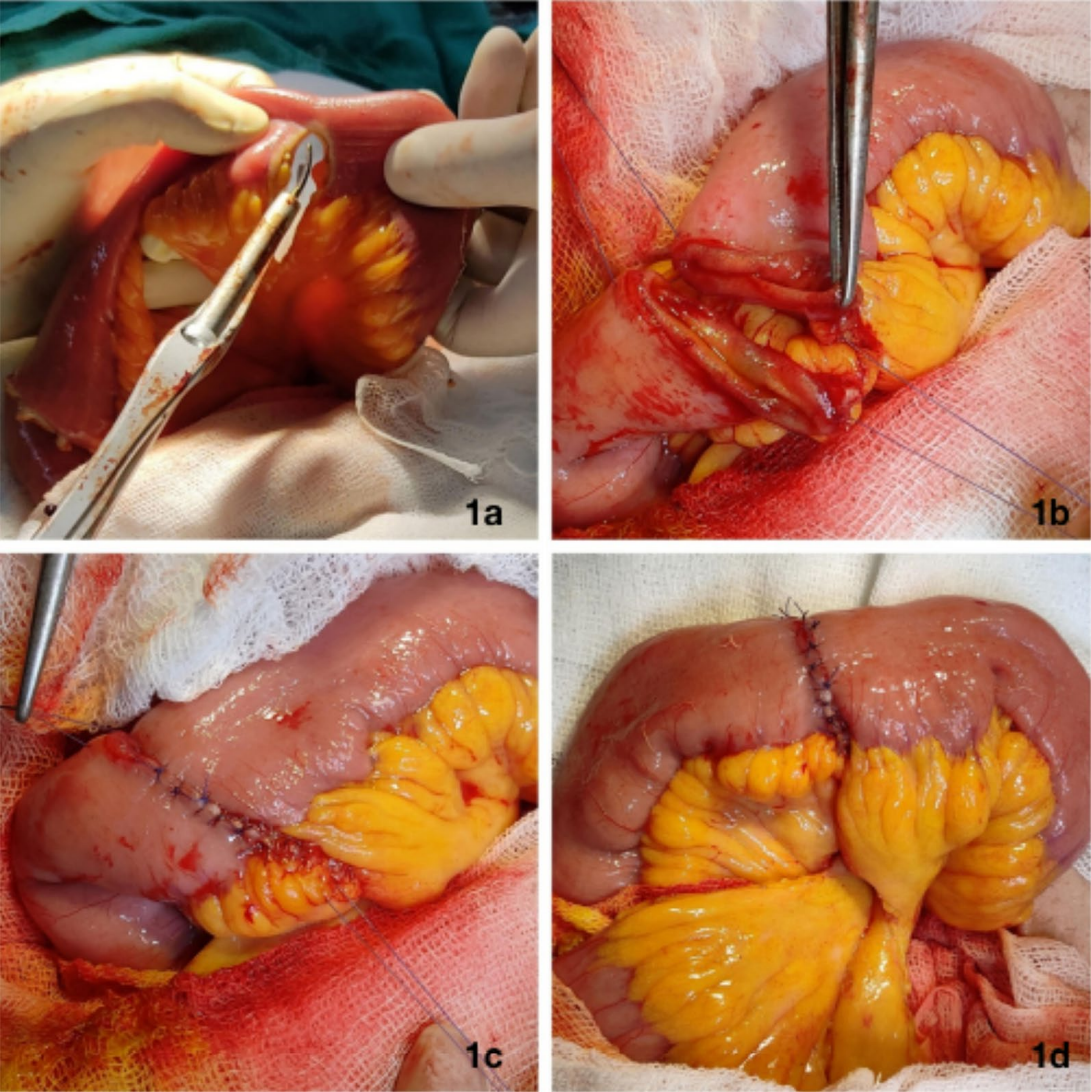
Intra-operative image showing extra-mucosal single interrupted layer anastomosis for re-approximating ileum. **1a** Division of ileal segment along with its mesentery for use in reconstruction using harmonic scalpel. **1b** Two Connell sutures (4–0 vicryl) placed at mesenteric and anti-mesenteric borders of aligned segments of ileum to be reapproximated. **1c** Interrupted sutures being placed 2 mm apart extramucosally on posterior and anterior walls to appose serosa of the ileal segments together. **1d** completed single layer interrupted ileoileal anastomosis

Excessive gut handling was minimized by using a harmonic scalpel (Fig. 1a). The mesenteries of the two ileal segments to be anastomosed were aligned, and two Connell sutures were placed on the mesenteric and antimesenteric sides of the aligned ileal segments using 4–0 Vicryl (Fig. 1b) sutures. Then, interrupted 4–0 Vicryl sutures were placed 2 mm apart on the posterior followed by anterior walls (Fig. 1c, 1d). On approaching the anti-mesenteric Connell suture, several sutures were placed before all were tied. A patent anastomosis was confirmed by feeling the annulus with the thumb and forefinger. All uretero-enteric anastomoses were stented, then removed after 2 weeks in IC and 4 weeks for patients with ONB.

Pathological stage, margin status, number of lymph nodes removed, and node density were assessed. The American Joint Committee on Cancer tumor, node, and metastases system was used for staging bladder cancer. Patients were followed weekly in the first month, then monthly for the next 2 months, and then followed up at 3 months post-surgery. The modified Clavien–Dindo classification was used to grade postoperative complications for a minimum follow-up period of 3 months [8].

## Statistical analysis

A statistical analysis was conducted using SPSS Statistics for Windows version 25.0, released in 2017 (IBM Corp., New York, USA). Data are expressed as a mean ± standard deviation (SD) or median (interquartile range) as apposite for continuous variables, whereas frequency and percentage are used to express qualitative data. Comparisons between groups were tested for significance using a chi-squared test for categorical variables and multiple ANOVA or Kruskal–Wallis test for continuous variables, as appropriate. All tests were two-sided. A *p*-value of < 0.05 was defined as statistically significant.

## Results

Among 59 patients who underwent ORC at our hospital during the study period, 49 had an IC procedure and 10 had an ONB. The overall median age was 53.35 ± 12.85 years: 52 ± 12.2 years for the IC group and 62.8 ± 12.04 years for the ONB group. The male to female ratio is 7.4:1. Fifty-one (86.4%) patients were from rural areas, and 48 (81.35%) were smokers. Fifty-six (94.9%) patients presented with painless gross hematuria, whereas three (5.08%) were diagnosed incidentally. Furthermore, 30 (50.84%) patients had hydroureteronephrosis due to infiltration of vesico-ureteric junction by the tumor and 23 (38.98%) had obstructive uropathy at the time of presentation. Percutaneous nephrostomy was done preoperatively in 15 (25.42%) patients in view of obstructive uropathy or hydronephrosis. The mean American Society of Anesthesiologists score of our study cohort was 1.9 ± 0.69, with a statistically significant difference between the two groups: 1.91 ± 0.64 in the IC group and 1.6 ± 0.84 in the ONB group (*p* = 0.015). Table 1 summarizes these findings.

**Table 1:** Demographic details of the patients undergoing radical cystectomy with urinary diversion

Parameter	Overall (n = 59)	Conduit (n = 49)	Orthotopic Neobladder (n = 10)	*p* value
Mean age (± SD), years	53.35(12.8)	52(12.2)	62.8(12.04)	0.125
*Gender*				
Male	52(88.1%)	44(89.8%)	8(80%)	**0.001**
Female	7(11.86%)	5(10.2%)	2(20)	
*Presentation*				
Hematuria	56(94.9)	47(95.9%)	9(90%)	0.673
Incidental	3(5.08%)	2(4.1%)	1(10%)	
Patients having obstructive uropathy at presentation	23(38.98%)	21(42.85%)	2(20%)	0.458
Patients undergoing preoperative percutaneous nephrostomy	15(25.42%)	14(23.72%)	1(10%)	0.501
ASA score, mean (± SD)	1.9 (0.69)	1.91(0.64)	1.6(0.84)	**0.015**
*Co-morbidities*				
Hypertension	25(42.3%)	18(36.7%)	6(60%)	0.381
Diabetes	18(30.50%)	12(24.5%)	5(50%)	0.266
Hypothyroidism	10(16.94%)	5(10.2%)	3(30%)	**0.015**
Old H/O Coronary artery disease	5(8.47%)	2(4.1%)	2(20%)	0.062
COPD	7(11.86%)	4(8.2%)	2(20%)	0.260
Presence of hydronephrosis on CT	30(50.84%)	25(51.02%)	5(50%)	0.797
*Dwelling*				
Rural	51(86.44%)	43(87.8%)	5(50%)	**0.012**
urban	8(13.55%)	6(12.2%)	5(50%)	
*Smoking Status*				
Smokers	48(81.35%)	36(73.5%)	10(100%)	0.169
Non Smokers	11(18.64%)	13(26.5%)	0(-)	
*Pesticide exposure*				
Yes	36(61.01%)	30(61.2%)	3(30%)	0.061
No	23(38.9%)	19(38.8%)	7(70%)	
Presence of significant pelvic lymphadenopathy on CT scan	28(47.45%)	24(48.97%)	4(40%)	0.132
Neo-adjuvant chemotherapy	8(13.56%)	8(16.3%)	0(-)	0.296

Values in bold are statistically significant parametres

*p < 0.05 considered as significant

ASA, American Society of Anesthesiologists Score; COPD, chronic obstructive pulmonary disease; CT scan, computed tomography scan

Transurethral resection of bladder tumor was done in all patients before planning the radical cystectomy. Preoperatively, 35 (59.32%) patients had muscle invasion on histopathology, and 23 (38.98%) had high-grade transitional cell carcinoma refractory to endoscopic or intravesical treatment. Eight (13.56%) patients received cisplatin-based neoadjuvant chemotherapy (three cycles). The mean operative duration was 263.8 ± 48.9 min, which was longer for the ONB group, compared with the IC group (278 ± 45.9 vs. 264.1 ± 49.2 min, *p* = 0.147), as expected. The mean time to perform the EMSIL ileoileal anastomosis was 17.3 ± 5 min, which was slightly but not significantly longer for patients undergoing ONB (17.9 ± 6 min), compared to IC (16.9 ± 4 min). See Table 2 for a summary.

**Table 2:** Pathological characteristics and stage of the patients undergoing radical cystectomy with urinary diversion

Parameter		Overall (n = 59)	Percentage
Preoperative histopathology	T2 disease (muscle-invasive high-grade TCC), n (%)	35	59.32
	High-grade TCC not amenable to endoscopic/intravesical treatment, n (%)	23	38.98
	High-grade TCC with squamous differentiation, n (%)	7	11.86
	High-grade TCC with sarcomatoid differentiation, n (%)	3	5.08
	Adenocarcinoma of bladder, n (%)	2	3.38
Pathological stage	pT1, n%	7	11.86
	pT2a, n (%)	11	18.64
	pT2b, n (%)	29	49.15
	pT3a, n(%)	9	15.25
	pT3b, n(%)	1	1.69
	pT4a, n(%)	2	3.38
	pT4b, n (%)	0(-)	0
Lymph nodal disease	pN0, n (%)	42	71.18
	pN1, n (%)	2	3.38
	pN2, n (%)	15	25.42
	pN3, n (%)	0(-)	0
Overall Complications	Minor complications(Grade 1 + 2)	9	15.25
	Major complications (Grade 3 + 4 + 5)	14	23.73
GI-Related Complications	Post-operative ileus (POI)	8	13.55
	NG-tube placement	5	8.47
	Total Parenteral Nutrition (TPN)	3	5.08
	Nausea	6	10.17
	Admission due to GI-related complications	9	15.25

TCC = transitional cell carcinoma

One patient (1.7%) underwent a urethrectomy for prostatic urethral involvement. Intra-operatively, one patient (1.7%) suffered a rectal injury, which was repaired primarily. The intraoperative mean blood loss was 721.3 ± 206.7 mL and similar in both groups. Thirty-eight (64.41%) patients needed blood transfusion (0.97 ± 0.79 units per patient). Patients in the ONB group required more peri-operative blood units (1.6 ± 0.52) than those in the IC group (0.88 ± 0.78), a significant difference between the two groups (p = 0.034). The mean duration (± SD) of post operative hospital stay was 12.9 ± 3.1 days, with a slightly longer stay for the ONB patient group (*p* = 0.480). Four (6.78%) patients needed post-operative ICU care (mean duration of 22.4 ± 47.2 h). The mean time to return of bowel sounds was similar between both groups. The average time to start oral intake was 84.6 ± 10 h. See Table 3 for a summary.

**Table 3:** Perioperative parameters of the patients undergoing radical cystectomy with urinary diversion

Parameter	Overall (n = 59)	Conduit (n = 49)	ONB (n = 10)	p value
Mean operative time in minutes ± SD	263 ± 48.9	264.1 ± 49.2	278 ± 45.9	0.147
Mean anastomosis time in minutes ± SD	17.3 ± 05	16.9 ± 04	17.9 ± 06	0.121
Mean intraoperative blood loss in ml ± SD	721.3 ± 206.7	694.3 ± 201.7	860 ± 195.5	0.422
Mean number of pRBC units transfused in peri-operative ± SD	0.97 ± 0.79	0.88 ± 0.78	1.6 ± 0.52	0.034
Mean duration of post operative ICU admission in hours ± SD	22.4 ± 47.2	20.6 ± 50.2	33.6 ± 36.85	0.607
Duration of abdominal drain in situ in days ± SD	6.4 ± 2.1	5.8 ± 2.7	6.1 ± 2.2	0.112
Mean duration of hospital stay in days ± SD	12.9 ± 3.1	12.7 ± 3.05	13.3 ± 3.6	0.480

Major complications (Grades III–V) were observed in 14 (23.73%) patients in the post-operative period which included wound dehiscence (10.67%), postoperative abdominal collection with septicemia (4.81%), and hospital-acquired pneumonia requiring ICU care (8.25%). One patient was re-explored for fecal peritonitis secondary to intestinal gangrene in the distribution of a branch of the superior mesenteric artery on day 10 after surgery and later on died due to septic shock. Table 4 summarizes the results. Ten (16.9%) patients developed at least one gastrointestinal complication during the first 90 days following ORC. Most gastrointestinal complications were minor (grade 1 = 46%, grade 2 = 54%). There were no major gastrointestinal complications in our study. The most common GI complication was postoperative ileus in eight (13.56%) patients. Only five (8.47%) patients needed a nasogastric tube, and total parenteral nutrition was required in only three. Other important GI complications included intractable nausea and vomiting (not enough to attribute to postoperative ileus) in six (10.16%) patients. Overall, nine (15.25%) patients required readmission for management of gastrointestinal complications during the 90-day follow-up period. The overall 90-day mortality rate was 3.39% (2 out of 59 patients). Among the deceased, one had dyselectrolytemia (predominantly hypokalemia and hyponatremia) due to sepsis secondary to fecal peritonitis. The other patient died of COVID-19 pneumonia.

**Table 4:** Comparison with major contemporary studies in Indian Population

Parameters	Current study	Gupta et al. [6]	Patidar et al. [7]	Nayak B et al. [20]
Total number of cases (N)	62	502	212	195
Ileal conduit (IC)	49	346	88	172
Orthotopic neobladder (ONB)	10	47	113	6
Operative time	263 ± 48	NA	300	303 ± 57
Intraoperative blood loss	721.3 ± 206	NA	700(400–1100)	977.5 ± 340
Average no. of blood units transfused	0.97 ± 0.79	NA	NA	2.3 ± 0.8
Mean hospital stay(days)	12.9 ± 3.1	NA	14.76 ± 7.71	10.4 ± 5.3
Overall Complication rate	37.09	25.7	64.1	49.3
Major complication rate	22.58	25	28	23.6%
GI-related complications	16.1	19.4	18	30.3

## Discussion

Postoperative period after radical cystectomy is usually eventful due to presence of comorbidities and poor performance. Our study analyzes outcome of single layer interrupted extra-mucosal ileo-ileal anastomosis for maintaining bowel continuity following ORC with urinary diversion.

Focusing on oncologic outcomes, no compelling evidence suggests superiority of either minimally invasive surgery or ORC [9]. Robotic radical cystectomy and ORC have similar oncological outcomes in terms of lymph node yield, positive margins, and short-term mortality. The only advantage of robotic cystectomy over ORC is less blood loss at the cost of more operative time [10]. In our study, the average operative duration was 263 ± 48.9 min, considerably shorter than the 348 min reported in a study of 63 patients who underwent robotic radical cystectomy [11]. On the contrary, a much prolonged mean operative time of 6.1 ± 1.3 h (5.8–6.5 h), mainly depending on the type of diversion, was reported in a large series of 516 patients who underwent ORC [12].

In our study, the average number of blood units transfused perioperatively was significantly less (0.97 ± 0.79) than that reported (1.4 ± 1.4) for a series of patients who underwent robotic radical cystectomy [11]. Also, the mean postoperative hospital stay in our patients was comparatively shorter, compared to that reported for robotic radical cystectomy cases (12.9 ± 3.1 days vs. 17.4 ± 4.7 days)[11].

The time required to prepare the bowel for anastomosis is considerably longer in cases of double-layer versus single-layer anastomosis. Meticulous circumferential clearing of the mesentery and omentum is required for the double-layer anastomosis to accommodate construction of the second intervening layer, where virtually no circumferential clearance is necessary for the single-layer method. A greater length of bowel wall (1 cm) is needed to apply the two layers of sutures, compared to the single layer. Additionally, time-related factors might influence the success of the single-layer method over double-layer methods, such as using only one layer of stitches, which consumes less time and has less lumen constriction.

Our study demonstrated a significantly lower mean anastomosis time of 17.3 ± 5 min, compared to the 36.7 ± 1.93 min and 30.7 ± 1.47 min reported for double-layer bowel anastomosis [13, 14]. Similar anastomosis times have been reported in studies across the globe [15, 16]. Furthermore, the EMSIL bowel anastomosis technique employed in our study aided in the early return of bowel sounds within 84.6 ± 10 h, significantly shorter than results reported in other studies employing similar surgical techniques [17]. Moreover, the EMSIL anastomosis in our patient cohort had certain advantages, such as improved nutritional status and faster recovery owing to early start of oral feeding.

In today’s cost-conscious environment, the price of staples for anastomosis is exorbitant compared to hand-sewn anastomosis, which can also be safely used in a similar time interval. We noted a considerable difference in the cost of materials for the ORC with hand-sewn EMSIL ileo-ileal anastomosis, compared to stapled or double-layer anastomosis. Only two flaps of Vicryl 4–0 absorbable violet braided sutures (45 cm each) were used for each EMSIL anastomosis, at a cost of about 850 Indian rupees. This cost would be at least twice as high for double-layer anastomoses and 20 times more for ORC with an EMSIL ileo-ileal anastomosis using staples.

The overall 90-day perioperative complication rate in our study was 38.98%, which was better than the 64% and 50% observed in similar studies in patient cohorts of different ethnicities [7, 19]. The results for complications were satisfactory when compared with contemporary studies from the Indian subcontinent. Furthermore, another study reported major complications in 41 out of 160 (25.5%) patients, including septicemia (3.7%), wound dehiscence (3.7%), post-operative ileus (2.5%), enterocolitis (1.2%), pulmonary embolism (1.2%), and peritonitis (0.6%), with a reoperation rate of 8.7% [18, 19]. The most common complications after radical cystectomy are GI-related with a frequency of approximately 35% [20, 21].

The ultimate test for the safety and efficacy of a technique for intestinal anastomosis is its rate of anastomotic leakage. In our study, no anastomotic leak or any other major GI complication occurred in any patients. Apart from generalized sympathetic hyperactivity resulting in depression of GI-motility, the surgical manipulation of abdominal contents is known to cause peristaltic impairment of the gut. Single-layer techniques of anastomosis thus are preferred, as they preserve the blood supply and full lumen width better than a double-layer technique [22]. Additionally, mucosal inversion has been proven superior to mucosal eversion in experimental and clinical studies. The rate of postoperative ileus in our technique was significantly lower at 13.55%, compared to the 23% documented in the literature for conventional double-layer bowel anastomosis after radical cystectomy [23]. In contrast, a postoperative ileus rate of only 5% was reported in a study using an interrupted single-layer technique for bowel anastomosis [24]. The reason for such a low incidence was the small sample size (21 patients) and the choice of urinary diversion (IC in all patients). In the present study, gut gangrene was observed in one patient, possibly due to the patient’s underlying cardiovascular comorbidities with consequent hypercoagulability and embolus seeding in an unnamed branch of the superior mesenteric artery.

Neoadjuvant therapy was underutilized in our study, similar to what is practiced even in Western countries, because most patients did not consent to this modality of management. Only eight out of 50 eligible patients consented to neoadjuvant chemotherapy in our series despite its proven benefits in randomized controlled trials. Currently NCCN as well as EUA guidelines recommend neoadjuvant chemotherapy for muscle invasive disease. However, concerns about age, comorbidities, and surgery delay are the primary deterrents for its use.

After evaluating the results of our analysis, several strengths should be highlighted. The study demonstrated the outcomes of ORC in the present era of minimally invasive surgery in a prospective manner. Few studies have analyzed 3-month perioperative complications of ORC in Indian populations. This study provides insight into the use of newer techniques and innovations in ORC in resource-poor countries without using staples. All operations were performed by a single surgeon, which eliminated any bias caused by differences in surgical expertise.

The downsides of our study were the small sample size and lack of comparison with minimally invasive radical cystectomy. The postoperative follow-up protocol and length of hospital stay also were weaknesses of our study. The postoperative pathway in our study is not commonly used by most high-volume centers, which favor protocols involving enhanced recovery after surgery.

## Conclusions

Extra-mucosal single layer ileo-ileal anastomosis for bowel re- approximation is safe and is associated with acceptable and easily manageable intestinal complications in patients following radical cystectomy and urinary diversion. In addition to short anastomosis time and rapid return of bowel activity, no major intestinal complication was observed in our patients. We advocate EMSIL as a viable choice for bowel continuity in urinary diversions following cystectomy.

## Data Availability

The datasets generated and/or analyzed during the current study are not publically available because it could compromise individual privacy but can be made available from the corresponding author on reasonable request.

## Abbreviations

EMSIL: Extra-mucosal single interrupted layer
IC: Ileal conduit
ONB: Orthotopic neobladder
ORC: Open radical cystectomy
POI: Post operative ileus
TCC: Transitional cell carcinoma
TPN: Total parenteral nutrition
